# ChloroSeq, an Optimized Chloroplast RNA-Seq Bioinformatic Pipeline, Reveals Remodeling of the Organellar Transcriptome Under Heat Stress

**DOI:** 10.1534/g3.116.030783

**Published:** 2016-07-06

**Authors:** Benoît Castandet, Amber M. Hotto, Susan R. Strickler, David B. Stern

**Affiliations:** Boyce Thompson Institute, Ithaca, New York 14853-1801

**Keywords:** *Arabidopsis thaliana*, chloroplast, RNA-Seq, heat stress, transcriptome, noncoding RNAs, introns, RNA splicing, RNA editing

## Abstract

Although RNA-Seq has revolutionized transcript analysis, organellar transcriptomes are rarely assessed even when present in published datasets. Here, we describe the development and application of a rapid and convenient method, ChloroSeq, to delineate qualitative and quantitative features of chloroplast RNA metabolism from strand-specific RNA-Seq datasets, including processing, editing, splicing, and relative transcript abundance. The use of a single experiment to analyze systematically chloroplast transcript maturation and abundance is of particular interest due to frequent pleiotropic effects observed in mutants that affect chloroplast gene expression and/or photosynthesis. To illustrate its utility, ChloroSeq was applied to published RNA-Seq datasets derived from *Arabidopsis thaliana* grown under control and abiotic stress conditions, where the organellar transcriptome had not been examined. The most appreciable effects were found for heat stress, which induces a global reduction in splicing and editing efficiency, and leads to increased abundance of chloroplast transcripts, including genic, intergenic, and antisense transcripts. Moreover, by concomitantly analyzing nuclear transcripts that encode chloroplast gene expression regulators from the same libraries, we demonstrate the possibility of achieving a holistic understanding of the nucleus-organelle system. ChloroSeq thus represents a unique method for streamlining RNA-Seq data interpretation of the chloroplast transcriptome and its regulators.

Expression of the chloroplast genome is the result of a complex interplay of nuclear and organelle-encoded proteins. As many as three RNA polymerases and six sigma factors create the primary transcriptome, which is further processed by an array of ribonucleases, RNA-binding proteins, and RNA splicing and editing factors, ultimately producing mature transfer RNAs, ribosomal RNAs, and messenger RNAs (tRNAs, rRNAs, and mRNAs, respectively) (Barkan 2011; Germain *et al.* 2013; Stern *et al.* 2010). Disruption of any of these posttranscriptional steps, whether initially affecting one or multiple genes, often leads to pleiotropic effects. These may be at the whole-plant level, such as chlorosis due to photosynthetic deficiency, and/or at the level of gene expression, for example because of global transcriptional or translational impairment. Therefore, most stresses and mutations affecting chloroplast gene expression warrant analysis at the genome rather than gene-by-gene scale.

The first global strategies for chloroplast transcript analysis utilized microarrays, most recently tiling arrays, which have yielded breakthroughs in our understanding of RNA targets of specific regulatory factors, developmental variations in the transcriptome, and more recently translation (Allorent *et al.* 2013; Nagashima *et al.* 2004; Schmitz-Linneweber *et al.* 2005; Zoschke *et al.* 2013). On the other hand, they have limited value as quantitative tools, are rarely developed to cover both strands of an entire genome, and do not reveal features such as splicing and editing status (Cahoon *et al.* 2008; Kahlau and Bock 2008; Nagashima *et al.* 2004; Nakamura *et al.* 2003; Schmitz-Linneweber *et al.* 2005; Valkov *et al.* 2009; Zmienko *et al.* 2011; Zoschke *et al.* 2013). These limitations have been largely overcome by high-throughput cDNA sequencing (RNA-Seq), which has rapidly become preeminent for plants since its initial application to *Arabidopsis thaliana* (Weber *et al.* 2007), and now for many model and nonmodel organisms (Martin *et al.* 2013). Currently, some 30,000 RNA-Seq libraries are archived for the Streptophyta taxon in the Sequence Read Archive (SRA) database (www.ncbi.nlm.nih.gov.sra).

RNA-Seq libraries are generally obtained from total RNA, and as a significant proportion of plant cellular RNA comes from organelles (Loening and Ingle 1967), they can be an invaluable resource to study chloroplast and mitochondrial RNA metabolism (Smith 2013). The major caveat is that most RNA-Seq libraries rely on enrichment of polyadenylated transcripts, representing mRNAs. Since plant organellar transcripts become unstable upon polyadenylation (Rorbach *et al.* 2014), library preparation protocols developed for organelles cannot use such a step. Fortunately, plant organelle-friendly libraries are becoming more prevalent, for example through work specifically designed to include organellar transcripts (Hotto *et al.* 2015, 2011; Zhelyazkova *et al.* 2012b), or to analyze nuclear noncoding RNAs (Di *et al.* 2014). Additionally, previously published RNA-Seq data can often be used to gain information aside from the original aim (Castandet *et al.* 2013; Ruwe and Schmitz-Linneweber 2012; Zhelyazkova *et al.* 2012a). Thus, published and novel RNA-Seq data can be harnessed to increase our understanding of how the chloroplast transcriptome responds to stress, genotype, and developmental factors.

In spite of the growing opportunities to discover properties of plant organellar transcriptomes, most compatible datasets have not been mined for their organellar component, in part because most of the analytical pipelines developed for nuclear transcriptomes are not adapted to the peculiarities of chloroplast gene expression. To facilitate cell-wide transcriptomic analyses, we developed ChloroSeq, a pipeline yielding a comprehensive view of chloroplast RNA processing, editing, splicing, and relative abundance. As applied to published data on *Arabidopsis* stress responses (Di *et al.* 2014), ChloroSeq reveals substantial effects at various steps of chloroplast gene expression under heat stress, some of which could be validated using gene-by-gene approaches. In addition, integration of these results with the nuclear transcriptome revealed RNA abundance changes for chloroplast-targeted enzymes that may contribute to alterations of the chloroplast transcriptome in response to heat stress, opening the way to a better understanding of nucleus-organelle crosstalk.

## Materials and Methods

### Hardware and software

All analyses described here were performed on a 64-bit laptop running the Ubuntu 14.04 LTS operating system with 8 GB (gigabytes) of RAM, with the following software installed: Bowtie2 version 2.2.3 (http://bowtie-bio.sourceforge.net/index.shtml), SAMtools (http://www.htslib.org/), TopHat2 version 2.0.11 (http://ccb.jhu.edu/software/tophat/index.shtml), Cufflinks version 2.2.1 (http://cole-trapnell-lab.github.io/cufflinks/), and BED tools version 2.25.0 (https://github.com/arq5x/bedtools2).

RNA-Seq data (Accession SRP028304; Di *et al.* 2014) was downloaded from the SRA database (Kodama *et al.* 2012) (http://www.ncbi.nlm.nih.gov/sra?term=SRP028304), and then converted into the corresponding FastQ file using fastq-dump from the SRA toolkit (http://www.ncbi.nlm.nih.gov/Traces/sra/sra.cgi?view=toolkit_doc&f=fastq-dump). Reads were initially quality trimmed using the fastq-mcf software (https://code.google.com/p/ea-utils/wiki/FastqMcf), and only bases with a quality score higher than 30 and reads with a minimum length of 60 were retained.

Before starting, ChloroSeq and the accompanying annotation files must be downloaded (https://github.com/BenoitCastandet/chloroseq) and ungrouped in the working directory. The annotation files specify genomic intervals to be used by ChloroSeq (see below). The TAIR10_ChrC_files directory contents further expand in TAIR10_ChrC_bowtie2_index, and contain the indexed files for the chloroplast genome used during the alignment.

### Genome alignment and differential expression analysis

Alignment to the chloroplast genome was performed using TopHat2, allowing up to two alignments to account for the chloroplast inverted repeats (–g 2). The GFF file containing the gene coordinates and the indexed genome files used can be found in the TAIR10_ChrC_files and TAIR10_ChrC_files/TAIR10_ChrC_bowtie2_index directories, respectively, and the novel splice site discovery was disabled (–no-novel-juncs).

In addition, differential expression of nuclear genes was assayed following the step-by-step Tuxedo protocol using TopHat and Cuffdiff (Trapnell *et al.* 2012). We provide an additional file (Supplemental Material, File S1) containing the genes known to be involved in chloroplast RNA metabolism, to simplify the extraction of their expression values.

### Running ChloroSeq

ChloroSeq is made of four bash shell script files (.sh files) that prepare the read file for processing, then address coverage, splicing, and editing, and one Perl script (.pl file) that runs the pipeline and calls the bash shell scripts as needed. All of these files need to be made executable before use. Here is an overview of ChloroSeq:

chloroseq.pl [-h] -a <analysis> -b <accepted_hits.bam> -e <exon.gff3> -i <intron.gff3> -g <genome_size>
-n <name> -v <editing_sites.gff3> -f <fasta> -s <splice_sites.gff3> -k <keep_files>

To obtain details on running the pipeline and available options, the following command may be used:

perldoc chloroseq.pl

### Preparing the bam file

When the pipeline is first run, the shell script prep_bam.sh is called to index the bam file. The desired reads corresponding to the genome of interest (here ChrC) are then extracted from the aligned reads bam file. The resulting bam file is then used for all other steps in the pipeline. This gives ChloroSeq the capacity to extract and analyze any subgenome of interest (for example chloroplast or mitochondrion) when the alignment has been performed against the complete genome. One simply needs to know the size of the genome (-g option) and the name of the genome of interest (-n option). The name of the genome of interest must match the one used for alignment.

### Analysis 1: coverage

By choosing option analysis 1 (-a 1) in the chloroseq.pl pipeline, the get_coverage.sh script is called and produces four different outputs: (1) single nucleotide depth of coverage of the complete chloroplast genome, (2) genome coverage using a 100 nt window, where each window overlaps the next by 50 nt, and RPKM values for (3) exons and (4) introns. RPKM values are calculated according to the formula $RPKM=C\frac{Ri}{TiL}$ where *C* is a constant (*C* = 10^9^), *Ri* is the number of reads that intersect the region of interest, *Ti* is the total number of reads of the sample, and *L* is the length of the region.

Once completed, six text files containing the four different outputs are found in the working directory: counts, bam_name, nt_coverage.txt, window_coverage.txt, exon_rpkm.txt, and intron_rpkm.txt. Intermediate files created may be kept with the –k option and are named coverage_plus.csv, coverage_minus.csv, window_plus.csv, window_minus.csv, exp_exon.txt, and exp_intron.txt.

### Analysis 2: splicing efficiency

Analysis 2 calls the get_splicing_efficiency script and produces one output file that contains the coordinates, the number of spliced and unspliced reads, and the calculated splicing efficiency for each intron. Splicing efficiency (*SE*) was calculated according to the formula $SE=\frac{jR}{jR+\left(liR+riR\right)/2}$ where *jR* is the number of reads spanning the exon/exon junction, *liR* is the number of reads spanning the 5′ exon/intron boundary, and *riR* is the number of reads spanning the 3′ intron/exon boundary.

The output file is named splicing_efficiency.txt and is found in the current directory. Intermediate files are named splice_junctions.bam, spliced_coverage.txt, and unspliced_coverage.txt, and may be retained with the –k option.

### Analysis 3: editing efficiency

Analysis 3 calls the shell script get_editing_efficiency.sh. This step produces two output files, editing.pileup and editing_efficiency.txt. The latter contains the coordinates of known editing sites for the *Arabidopsis* chloroplast (Ruwe *et al.* 2013), the plus strand identity of the edited nucleotide (C if plus strand, G if minus strand), the total coverage of the editing site, and the coverage for each of the four possible nucleotides. If a gff3 file of known editing sites is provided (-v option), the ratio of edited to (unedited + edited) reads is calculated to obtain the editing efficiency. The script uses SAMtools to create a pileup file containing the variants (Li *et al.* 2009).

### Plant growth conditions

*A. thaliana* ecotype Columbia was grown according to Di *et al.* (2014). Briefly, seeds were plated on 1/2 MS agar (1% sucrose, 0.1% MES, pH 5.7, 1.2% agar) under sterile conditions, and, after vernalization, were grown at room temperature under a 16 hr/8 hr light/dark cycle with 100 µmol/m^2^/sec light. After 12 d, seedlings were exposed to 37° heat stress for 0, 3, or 12 hr. Seedlings were harvested in microfuge tubes, frozen in liquid nitrogen, and stored at -80°.

### RNA extraction and analysis

Total RNA was extracted using TRI reagent (Molecular Research Center http://mrcgene.com/) according to the manufacturer’s instructions. For RNA blot analysis, 1 or 5 µg of total RNA was separated in 1.2% agarose/formaldehyde gels, blotted overnight onto Hybond-N+ (GE Healthcare), and hybridized with either α-^32^P-dCTP-labeled double-stranded DNA or α-^32^P-UTP-labeled single-stranded RNA probes as previously described (Hotto *et al.* 2011). Amounts of RNA analyzed and type of probe used are indicated in the Figure Legends.

### Data availability

The RNA-Seq data used in this article was obtained from the SRA database (Accession SRP028304; http://www.ncbi.nlm.nih.gov/sra?term=SRP028304). All bioinformatic scripts developed for this project can be found on GitHub (https://github.com/BenoitCastandet/chloroseq).

## Results and Discussion

### ChloroSeq overview

**Figure 1:**
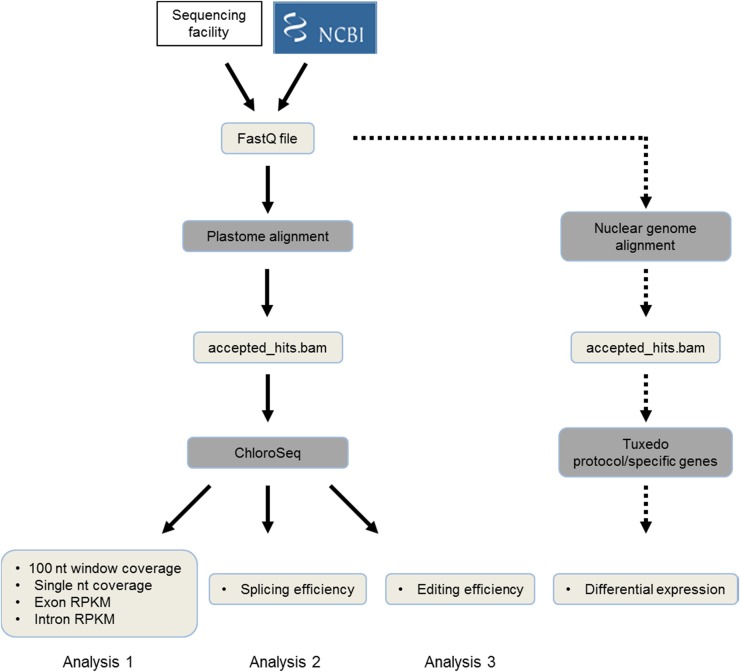
Flow chart of the ChloroSeq strategy. RNA-Seq reads (obtained from public databases or a sequencing facility) in the FastQ format are aligned to the plastome genome using TopHat2. ChloroSeq is then applied to the bam file containing aligned reads, giving a complete view of the chloroplast transcriptome. Using the same data, it is possible to concomitantly analyze the nuclear transcriptome using, for example, the Tuxedo protocol. NCBI, National Center for Biotechnology Information; nt, nucleotide; RNA-Seq, RNA sequencing; RPKM, reads per kilobase per million mapped reads.

Most published RNA-Seq libraries have only been used to study differential gene expression (DGE), and step-by-step protocols have been published to facilitate such analyses (Ghosh and Chan 2016; Trapnell *et al.* 2012). DGE does not, however, capture the range of transcriptome modifications that occur in organelles. For example, the widespread transcript abnormalities that occur in mutants lacking chloroplast polynucleotide phosphorylase (PNPase; (Castandet *et al.* 2013; Germain *et al.* 2011; Walter *et al.* 2002), spanning 3′ end maturation, editing, and splicing, cannot be identified by DGE. To overcome this limitation, we created ChloroSeq (https://github.com/BenoitCastandet/chloroseq), a pipeline designed to reveal these subtler, yet critical facets of chloroplast gene expression. The strategy is depicted in Figure 1, beginning with reads in the FastQ format from either a sequencing facility or downloaded from a database. The reads are then aligned to the chloroplast genome using TopHat2 (Kim *et al.* 2013). Following this, ChloroSeq extracts and counts the number of aligned reads (from the accepted_hit.bam file) that intersect the genomic intervals of interest, using the BEDTools suite (Quinlan 2014). Three different analyses can be completed with ChloroSeq, which can be run as a group or individually. Coverage analysis produces genome coverage data, including exon and intron Reads Per Kilobase per Million mapped reads (RPKM) information; splicing analysis generates splicing efficiency; and editing analysis yields RNA editing levels. The analyses all generate text files, which can be read by any statistical software to produce graphical representations of the data. An ancillary pipeline yields nuclear DGE. All the annotation files used here are based on the TAIR10 version of the *A. thaliana* genome, modified to add the first exon of the chloroplast gene *ycf3*. ChloroSeq has been optimized for single-end strand-specific RNA-Seq data, but can be adapted to work with paired-end reads. Additional details are given in *Materials and Methods*.

### rRNA subtraction increases chloroplast transcriptome coverage

Because rRNA constitutes the vast majority of cellular RNA, RNA-Seq library preparation protocols either selectively target poly(A+) RNA using oligo(dT) to prime cDNA synthesis, or deplete rRNA through hybridization to complementary oligonucleotides. Only the latter method preserves chloroplast transcripts, which are primarily free of poly(A) tails, and was applied to nearly 200 plant RNA-Seq libraries deposited in the SRA database. rRNA subtraction is becoming more widely used, however, because of the recognition that important components of the nuclear transcriptome are not polyadenylated, such as noncoding RNAs (ncRNAs) (Di *et al.* 2014).

The effect of rRNA depletion on the chloroplast genome alignment rate is illustrated in Table 1, where we compare RNA-Seq libraries prepared from poly(A)-depleted total RNA followed by rRNA subtraction (Group A); total RNA from isolated chloroplasts or seedlings, followed by rRNA subtraction (Group B); and an oligo(dT)-primed reverse transcription method (Group C). Approximately 30% of the reads aligned to the plastome from the rRNA-depleted libraries, whereas less than one percent of the oligo(dT)-selected RNAs could be aligned. When chloroplasts are purified before library preparation, the alignment rate reaches 75%, while young seedlings (2 d post emergence) contained less chloroplast RNA than older ones (12 d), on a total RNA basis. The low alignment rate for oligo(dT)-selected RNAs may mask quantitative and qualitative information, and place many transcripts below the limit of detection, skewing downstream gene expression analysis. On the other hand, such transcript pools could be useful for studying polyadenylation-mediated RNA degradation. In general, however, applying rRNA subtraction, without selection for poly(A)-tailed RNA, is essential for the comprehensive study of chloroplast RNA metabolism via RNA-Seq.

**Table 1:** Summary of chloroplast alignment rates for several published libraries

Name	Tissue	RNA Preparation	Mappable Reads	Mapped Reads	Mapped/Mappable (%)	Reference
Group A reads						
Control (SR63)	12-day-old seedlings	PolyA(−), rRNA subtraction	24057306	6088444	25.3	Di *et al.* 2014
Heat_3h (SR66)	12-day-old seedlings	PolyA(−), rRNA subtraction	36308522	10984310	30.3	Di *et al.* 2014
Heat_3h (SR67)	12-day-old seedlings	PolyA(−), rRNA subtraction	33484374	10205141	30.5	Di *et al.* 2014
Group B reads						
Col-0	Chloroplasts	Total, rRNA subtraction	12306665	9541442	77.5	Hotto *et al.* 2015
rnc3/4	Chloroplasts	Total, rRNA subtraction	13433730	9941425	74	Hotto *et al.* 2015
Col-0	2-day-old seedlings	Total, rRNA subtraction	26945257	3711755	13.8	Woodson *et al.* 2013
* gun1*	2-day-old seedlings	Total, rRNA subtraction	37022286	3823419	10.3	Woodson *et al.* 2013
Group C reads						
Col_15mn-1	5-day-old seedlings	Total, oligo(dT) RT	5067731	36746	0.7	Chen *et al.* 2015
Col_15mn-2	5-day-old seedlings	Total, oligo(dT) RT	4686444	36680	0.8	Chen *et al.* 2015
Col_15mn-3	5-day-old seedlings	Total, oligo(dT) RT	5133265	37018	0.7	Chen *et al.* 2015

In all cases, mappable reads represent reads with a quality higher than 30, and mapped reads represent the mappable reads that aligned to the *Arabidopsis* chloroplast genome (TAIR10) using TopHat2. Up to two locations were accepted for sequence reads to account for the large inverted repeat in the chloroplast genome. Group A reads from Di *et al.* (2014) were produced from poly(A) depleted total RNA followed by rRNA subtraction; Group B reads were obtained from total RNA from isolated chloroplasts or seedlings, followed by rRNA subtraction (Hotto *et al.* 2015; Woodson *et al.* 2013); and Group C reads were obtained following oligo(dT)-primed reverse transcription (Chen *et al.* 2015). Poly(A), poly(adenosine); rRNA, ribosomal RNA; oligo(dT) RT, oligo(dT)-primed reverse transcription.

### Coverage analysis

To give a rapid overview of the chloroplast transcriptional landscape from both DNA strands, ChloroSeq analysis 1 yields a 100 nucleotide (nt) window coverage overlapping by 50 nt, normalized to the total number of reads. Graphical representation of the window coverage, for example, can be used for quick identification of regions that merit further investigation or validation using molecular techniques. Additionally, a more precise view, for instance where coverage varies dramatically within a region, can be achieved using the single nucleotide coverage output. Importantly, either coverage analysis does not require any *a priori* knowledge of a region of interest, and in our experience is particularly valuable for the study of mutants or growth conditions whose effects are largely unknown. Such a strategy has already proven useful to identify a plastid noncoding (pnc) RNA specifically regulated by chloroplast Mini-ribonuclease III, and previously unidentified 3′ extensions and cleaved tRNA leaders found in the PNPase mutant (Castandet *et al.* 2013; Hotto *et al.* 2015).

**Figure 2:**
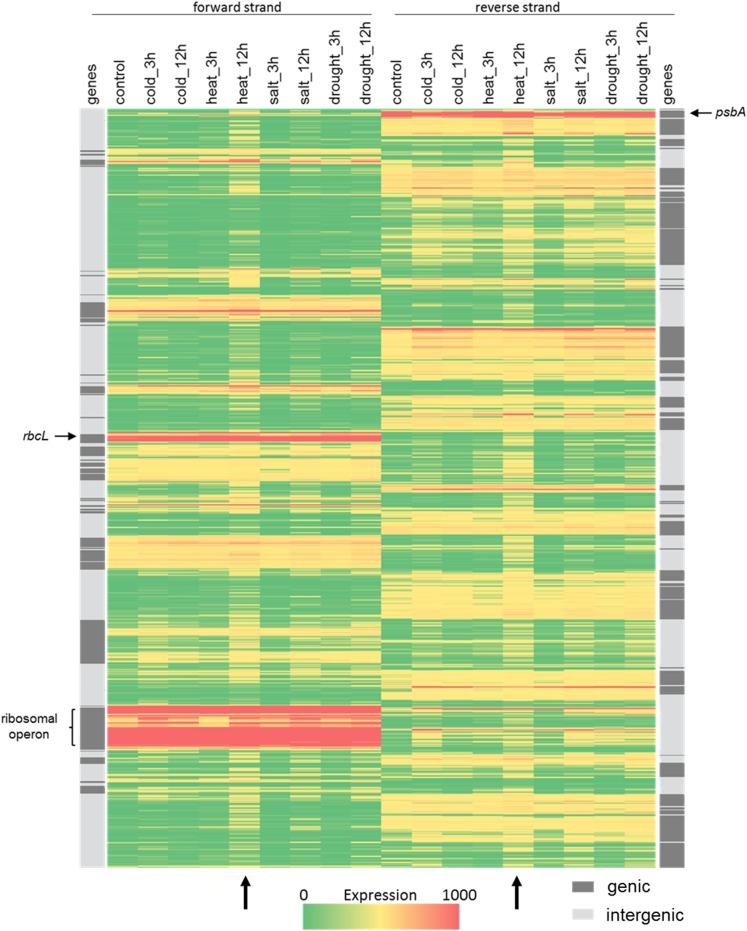
Heat map representation of chloroplast transcript levels following application of abiotic stresses. The window coverage data given by ChloroSeq analysis 1 was used to create the heat map. Low to high expression is represented by a green to red transition. Results are split between the forward (left) and reverse (right) strands. Dark gray stripes (outside lanes) represent known genic areas. The highly expressed *psbA* and *rbcL* genes, and the ribosomal operon are marked for reference. Vertical black arrows indicate the 12 hours heat stress treatment disscussed in the text.

In the present study, ChloroSeq 100 nt coverage was completed using RNA-Seq data from *A. thaliana* plants grown under control, heat, salt, cold, and drought stress conditions (Figure 2 and Figure S1). Figure S1 illustrates how the complete genome can be rapidly scanned by eye to reveal relative expression levels, while Figure 2 is a heat map representation of the same coverage. The heat map shows a strong correlation between RNA accumulation under control conditions, and the positions of annotated genes. The high expression of rRNAs, even after rRNA subtraction, is evidence that defects in rRNA processing can still be readily identified (Hotto *et al.* 2015). Comparison of heat maps between the control and four different stress conditions readily revealed that 12 hr of heat stress (vertical arrows at bottom) produced the strongest effect on the transcriptional landscape. We therefore focused our downstream analysis around the heat stress phenomenon.

### pncRNA levels increase under heat stress

**Figure 3:**
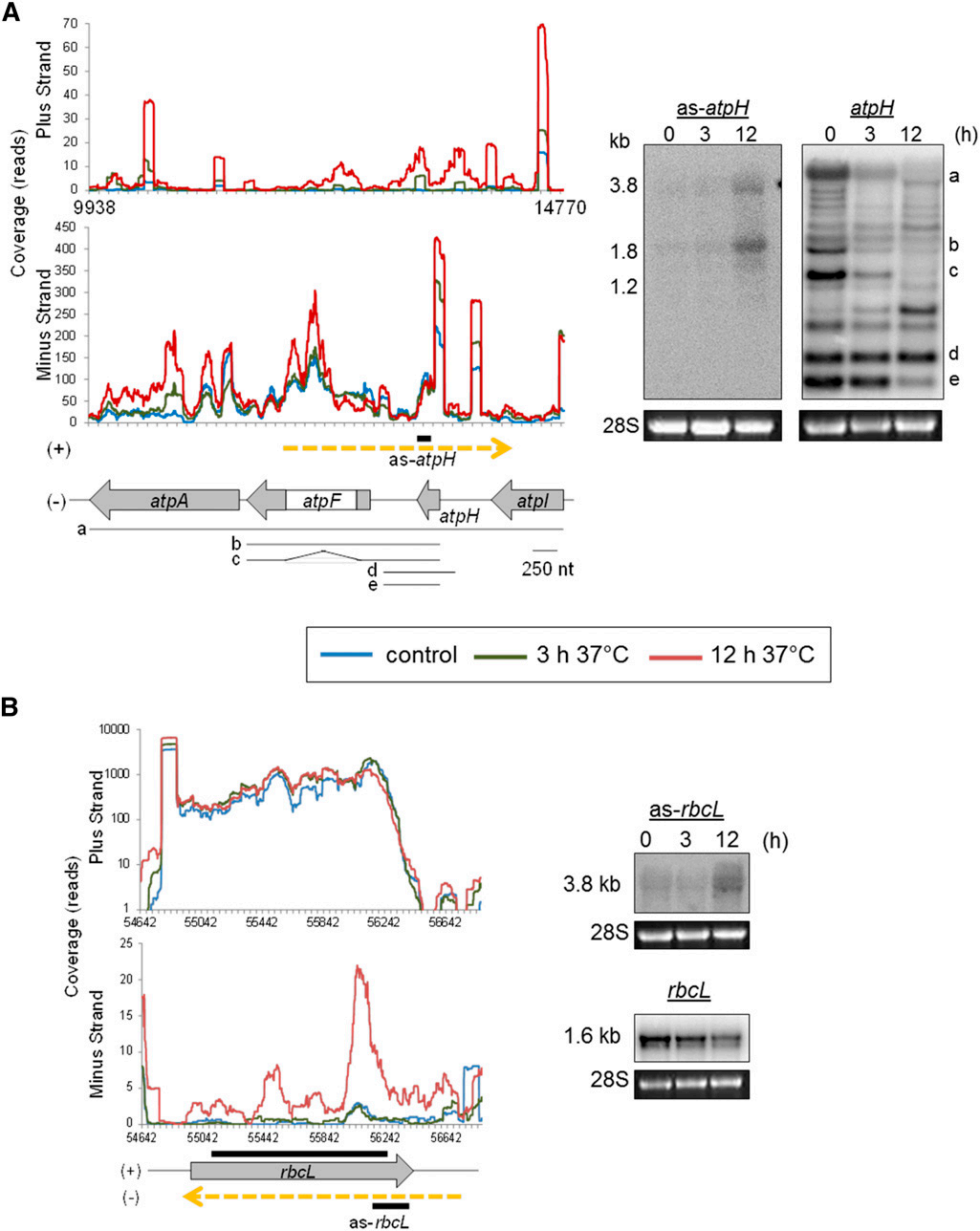
Accumulation of as-*atpH* and as-*rbcL* under heat stress. (A) Normalized RNA-Seq read coverage in the *atpH* coding region (left panel) for 0 (blue line), 3 (green line), or 12 hr (red line) at 37° for either the plus or minus strands, with the gene model shown below depicting the extent of sense (gray arrows) and antisense *atpH* transcripts (dotted yellow arrow). The thick black line represents the strand-specific RNA probe used for gel blot analysis (right panel) to detect either *as-atpH* or sense *atpH* with 5 µg of total RNA. The *atp* operon model shows the major *atpH*-containing transcripts (lettered a–e correlating to bands identified in Germain *et al.* 2011). Stained 28S rRNA is included to reflect loading and size markers are shown at left. (B) Analysis of the *rbcL* coding region by RNA-Seq and gel blot as described for (A). For the *rbcL* sense gel blot, 1 µg of RNA was analyzed using a double-stranded DNA probe. nt, nucleotide; RNA-Seq, RNA sequencing; rRNA, ribosomal RNA.

Earlier RNA-Seq analysis by ourselves and others had revealed the presence of more than 100 accumulating noncoding RNAs in chloroplasts, which have been termed pncRNAs (Hotto *et al.* 2012, 2011; Wang *et al.* 2011; Zhelyazkova *et al.* 2012b). An increase in the pncRNA population after 12 hr of heat stress could be recognized either by inspection of the 100 nt window coverage or heat maps. In the former case, numerous coverage peaks outside of genic regions became substantially higher upon heat stress (asterisks in Figure S1A). In the heat map, many areas outside the genic regions (light gray bars at edges of Figure 2) exhibited low expression (green color) under control conditions that increased under heat stress, as indicated by a shift toward yellow or orange hues. Variation in the pncRNA population was expected, as heat stress decreased pncRNA accumulation in *Brassica rapa* (Wang *et al.* 2011). The two studies are not directly comparable, however, due to differences in heat treatment (≥ 44° *vs.* 37°), type of pncRNA detected (< 36 nt, in contrast to our cutoff of > 100 nt for *Arabidopsis*), and species examined, but rather corroborates the influence of heat on pncRNA accumulation. To see whether apparent pncRNA increases would be reflected in RNA gel blots, we examined transcripts antisense to *atpH* (as-*atpH*; Figure 3A) and *rbcL* (as-*rbcL*; Figure 3B). In both cases, the pncRNA increased after 12 hr of heat stress, while little to no transcript was detectable at 0 and 3 hr of heat, confirming the RNA-Seq result.

RNA-Seq data plots for the *atpH* and *rbcL* coding regions exhibited minor differences following 12 hr of heat stress. The *rbcL* mRNA appeared to decline slightly by RNA gel blot, whereas the *atpH* transcript pattern changed qualitatively, due to altered transcript processing or stability within the polycistronic transcription unit. Such shifts are generally not visible in RNA-Seq profiles, and the findings in this instance illustrate certain strengths and limitations of each approach. Whether the observed increases in the pncRNAs are related to the alterations in sense transcripts cannot be determined, especially given the ∼10–40-fold differences in coverage levels when the two strands are compared.

**Figure 4:**
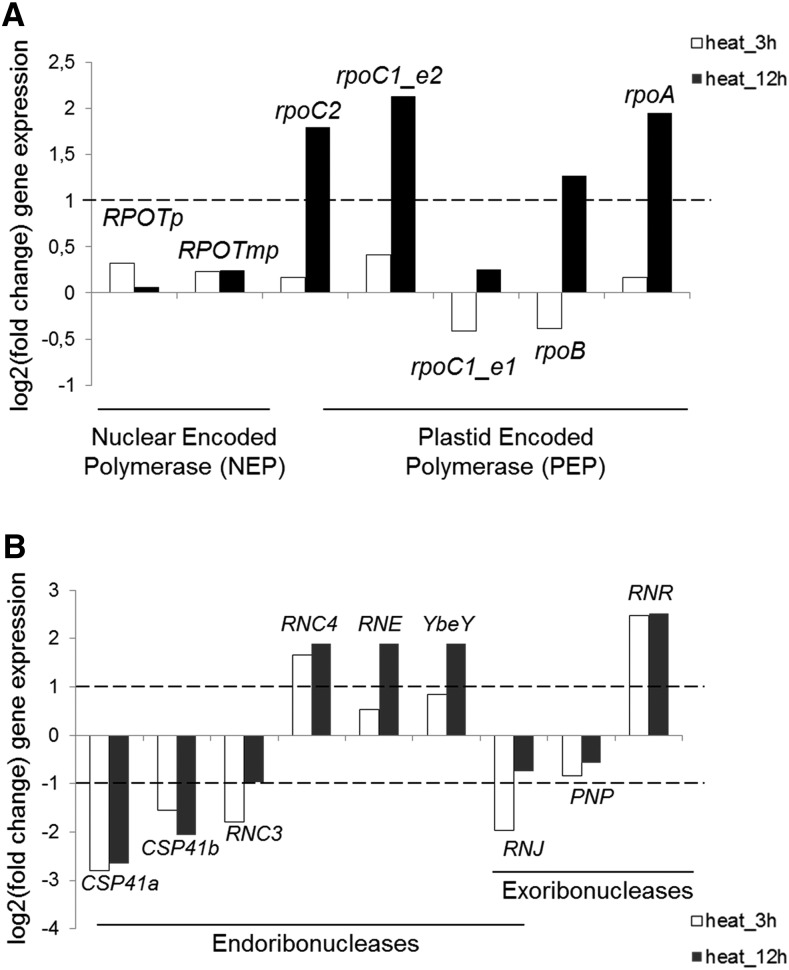
Accumulation of transcripts encoding the plastid transcriptional machinery and ribonucleases under heat stress. (A) Fold gene expression compared to the control is on the log2 scale for 3 hr (white bars) and 12 hr (black bars) of heat stress, with the dotted line representing a twofold difference. *RPOTp* and *RPOTmp* encode the phage-type RNA polymerases; *rpoA-C* encode the bacterial-like RNA polymerase core subunits. (B) The graph description is as in (A). *CSP41a* and *b* encode the paralogous chloroplast stem-loop binding proteins/ribonucleases CSP41a and CSP41b; *RNC3* and *RNC4* encode redundant mini-ribonuclease III proteins; *YbeY* encodes endoribonuclease Y; *RNJ* encodes ribonuclease J; *PNP* encodes polynucleotide phosphorylase; and *RNR* encodes ribonuclease II/R. NEP, nuclear encoded polymerase; PEP, plastid encoded polymerase.

The mechanism underlying pncRNA proliferation here may be the consequence of increased transcriptional activity and/or pncRNA stability. Little is known about the effect of heat stress on chloroplast transcription, apart from experiments in maize showing that chloroplasts were 5–10 times more transcriptionally active in plants grown at 37° for 24 hr compared to plants kept at 20° (Nakajima and Mulligan 2001). Here, the genes encoding components of the plastid-encoded RNA polymerase exhibited an increase after 12 hr of heat stress, suggesting that transcriptional activity may partially contribute to the increase in pncRNAs (Figure 4A). Differences in ribonuclease activity at elevated temperatures could also underlie altered pncRNA stability. Candidates would include RNase J, which prevents the overaccumulation of antisense RNAs (asRNAs), including an as-*rbcL* similar to the one observed here (Sharwood *et al.* 2011a), as well as PNPase, RNase II/RNR1, and Mini-III, which have all been shown to affect the pncRNA population (Sharwood *et al.* 2011b; Hotto *et al.* 2011, 2015). As shown in Figure 4B, transcripts from nuclear genes encoding chloroplast RNases exhibited both declines (CSP41, RNC3, and RNase J) and increases (RNC4, RNase E, YbeY, and RNase II/R) upon heat stress. In particular, the RNase J transcript decreased, raising the possibility that this could contribute to the accumulation of pncRNAs. However, possible heat effects on enzyme abundance and catalytic activity also need to be taken into account.

### Intronic and some exonic reads accumulate under heat stress

**Figure 5:**
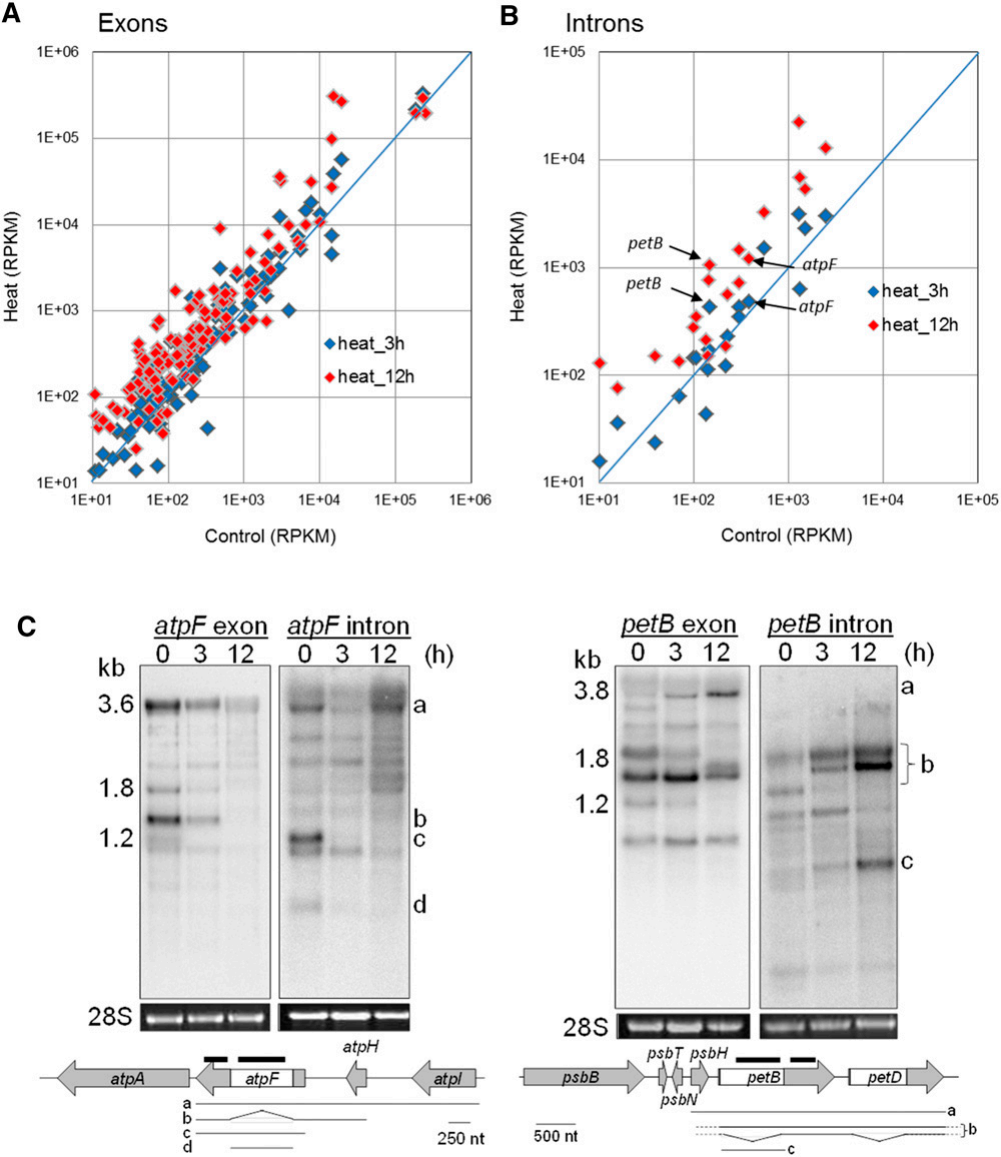
Increased intron accumulation under heat stress. Fold gene expression change of exons (A) and introns (B) between control and heat stress conditions by comparison of RPKM values. (C) RNA gel blot analysis of exons and introns for *atpF* (left) and *petB* (right) analyzed with double-stranded DNA probes (thick black bars in operon models) and 1 µg of total RNA. Letters on the right identify transcripts that are depicted in gene models under the blots. Stained 28S rRNA is included to reflect loading, and size markers are at the left. nt, nucleotide; RPKM, reads per kilobase per million mapped reads.

ChloroSeq analysis 1 also produces exon and intron RPKM values used to identify differential accumulation of reads for these features in the chloroplast. We first used a box plot to inspect the sum total of exon and intron values upon heat stress (Figure S2). Only after 12 hr of heat stress did total exonic RPKMs appear higher than the control condition, but the difference is not statistically significant (Figure S2A). However, this trend is amplified for introns, for which median RPKMs are approximately fivefold higher after 12 hr of heat stress compared to control and other stress conditions (Figure S2B). Examination of individual exons and introns revealed that at 12 hr of heat stress, a small number of exons appeared to be responsible for the overall RPKM increase (Figure 5A), whereas the majority of introns evinced increased accumulation under the same condition (Figure 5B).

The *atpF* and *petB* transcripts were further analyzed by RNA gel blot. For both introns, the transcript pattern changed dramatically after 12 hr of heat with a trend toward longer, unprocessed products. Quantitatively, more intron-containing transcripts were detected at 12 hr for *atpF* and *petB*, but less so for *atpF*, consistent with the RNA-Seq result (Figure 5C). Similarly, transcript processing for the corresponding exonic regions drifted toward unprocessed, longer transcripts, although quantitatively *atpF* seemed to decrease while *petB* appeared largely unchanged. Reduced processing would tend to increase intron-containing reads compared to those from exons, and could result from increased transcription overloading the splicing machinery. Reduced RNase activity might also play into the observed results.

### Heat stress impairs splicing

Splicing in the chloroplast was quantified using ChloroSeq analysis 2, which measures the ratio of spliced to total RNA for a given gene. In this case, splicing efficiency for the six tRNA introns in *Arabidopsis* cannot be calculated accurately, as the spliced tRNAs fall below the 100 nt threshold that was excluded from the library preparation protocol used (Di *et al.* 2014). However, other strand-specific libraries may have higher cutoffs that make them amenable to tRNA splicing analysis.

**Figure 6:**
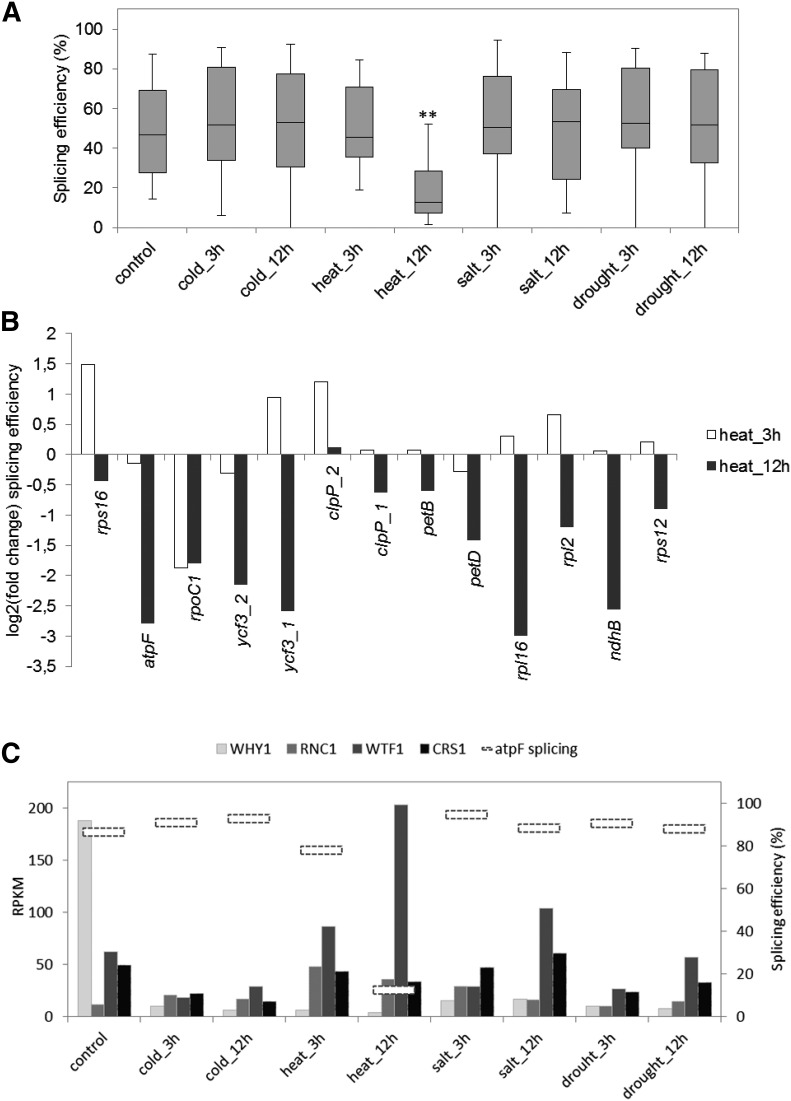
Heat stress inhibits splicing. (A) Box plot representation of splicing efficiencies under different stress conditions. The horizontal bars represent median splicing efficiencies and the top and bottom of the boxes represent 25 and 75% of the distribution, respectively. The top and bottom whiskers represent the highest and lowest efficiencies, respectively. The tRNA introns were omitted from the analysis because of poor coverage in this type of RNA-Seq library. ** *P* < 0.01 in a Student’s *t*-test. (B) Splicing efficiency change after 3 hr and 12 hr of heat stress as compared to control conditions. (C) Relationship between the splicing efficiency of the *atpF* intron (horizontal dashed boxes; Y2 axis) and expression of RNAs encoding protein factors involved in its splicing under different stress conditions (RPKM; Y1 axis). RNA-Seq, RNA sequencing; RPKM, reads per kilobase per million mapped reads.

When the effect of abiotic stress on collective chloroplast splicing apart from tRNAs was examined, a strong inhibition specific to heat stress could be identified (Figure 6A). Individually, all introns are less efficiently spliced after 12 hr of heat stress, with the exception of *clpP* intron 2 (Figure 6B). Heat-sensitive splicing was also documented for *ndhB* in tobacco (Karcher and Bock 2002), and for *ycf3* intron 1 in the barley CL3 mutant, where two point mutations in the intron were proposed to alter its secondary structure in a thermosensitive manner (Landau *et al.* 2009). Whether wild-type intron structures are sensitive to temperature in a functionally significant manner, or whether more rapid transcription impairs proper intron folding (reviewed by Schmitz-Linneweber and Barkan 2007), remains to be determined.

Akin to mechanisms underlying altered RNA accumulation, splicing *trans* factor expression could be related to splicing efficiency in response to heat stress. These nucleus-encoded factors (reviewed in Germain *et al.* 2013) include both gene-specific members and others that service multiple introns. To achieve the general splicing inhibition observed would connote a suppression of at least one factor required for the splicing of each intron, which was not observed at the RNA level (Figure S3A). This is well illustrated by the *atpF* intron, for which four proteins (WHY1, RNC1, WTF1, and CRS1) (Kroeger *et al.* 2009; Prikryl *et al.* 2008; Till *et al.* 2001; Watkins *et al.* 2007) are known to be involved in its splicing. Of these, only transcripts for WHY1 experience a drastic reduction under heat stress (Figure 6C). This reduction is, however, not specific to heat stress and therefore cannot explain by itself the *atpF* splicing inhibition. Although WTF1 expression shows an inverse correlation with *atpF* splicing under heat stress, reciprocity is not observed after 12 hr of salt stress. Similarly, the *CRS1* gene is expressed at a higher level in green tissues than in roots, correlating with *atpF* splicing, but is also highly expressed in the leaf base where splicing is less efficient (Till *et al.* 2001). Finally, the chloroplast-encoded splicing factor MatK, which is known to have a role in the removal of several introns (Zoschke *et al.* 2010), appears to be upregulated after 12hr of heat stress, in contrast to the general deficiency in splicing (Figure S3A). Therefore, the results to date do not support a direct link between expression at the RNA level of a given splicing factor and splicing efficiency.

### Heat stress and RNA editing

**Figure 7:**
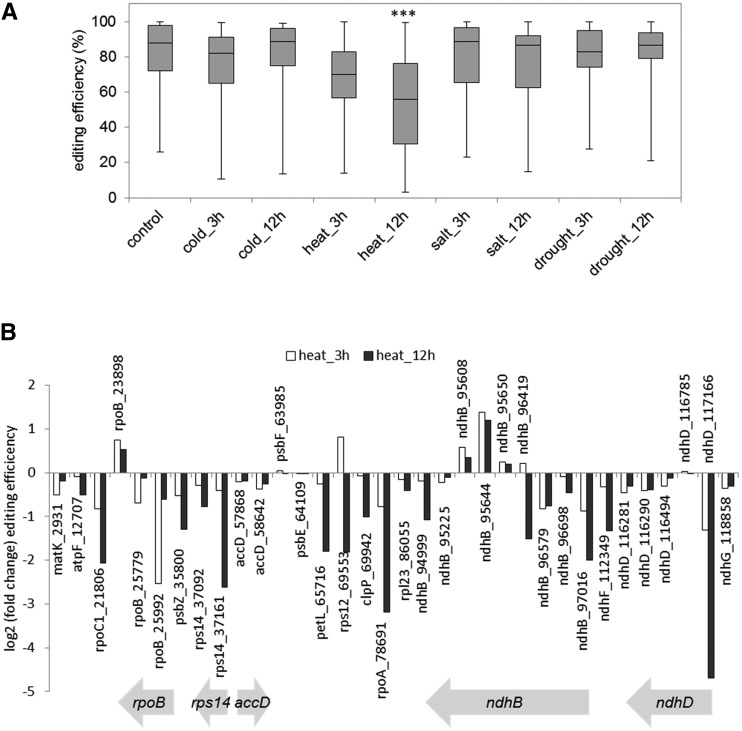
Heat stress inhibits RNA editing. (A) Box plot representation of editing efficiencies under different stress conditions. The horizontal bar represents the median editing efficiencies and the top and bottom of the boxes represent 25 and 75% of the distribution, respectively. The top and bottom whiskers represent the highest and lowest editing efficiencies, respectively. Recently-discovered editing sites (Ruwe *et al.* 2013) were not included in the analysis, because they are poorly edited even under control conditions. *** *P* < 0.001 in a Student’s *t*-test. (B) Change in editing efficiency after 3 hr and 12 hr of heat stress, compared to control conditions. Genes containing more than one editing site are indicated by gene models across the bottom.

Editing in the chloroplast was quantified using ChloroSeq analysis 3. Like splicing, editing showed a significant collective reduction after 12 hr of heat stress (Figure 7A). Editing was also reduced for most individual sites, although some displayed little variation in editing efficiency (*psbF*_63985, *psbE*_64109, *ndhD*_116785, *ndhB*_95225, or *rpl23*_86055), or modest increases (*rpoB*_23898, *ndhB*_95608, and *ndhB*_95644) (Figure 7B). Different editing sites within a single transcript sometimes displayed opposite behavior, as illustrated by *rpoB*, *ndhB*, and *ndhD*. The reduction of editing that we observed for *rps14* (Figure 7B) also occurs in heat-stressed maize (Nakajima and Mulligan 2001). Site-specific influence of heat on *ndhB* editing has been reported for tobacco (Karcher and Bock 1998, 2002), and our results are in partial concurrence. For example, editing sites 96579 (EII in tobacco) and 95650 (EVI) display the same behavior in both species, being heat-sensitive and insensitive, respectively, whereas site 95644 (EVII), which was described as heat-insensitive in tobacco (Karcher and Bock 2002), is increasingly edited upon heat stress in *Arabidopsis* (Figure 7B).

Some sites show a different behavior. Editing at *rpoB_*25992 displays a stronger reduction after 3 hr of heat stress compared to 12 hr, and the *rps12_69553* site experiences an increase in editing after 3 hr and a decrease after 12 hr. It is worth noting that two recently identified proteins involved in chloroplast RNA editing, RIP2 and RIP9 (Bentolila *et al.* 2013), show either an opposite behavior between 3 and 12 hr (RIP2 is first downregulated then upregulated) or a stronger downregulation after 3 hr than 12 hr (RIP9) (Figure S3B). In some cases, editing factor expression correlates with editing efficiency (Figure S3B). For example, CRR21 and OTP80 expression remained stable under heat stress, as did their corresponding editing sites *ndhD*_116785 and *rpl23*_86055. On the other hand, editing sites *rps14*_37161, *clpP*_69942, and *rpoA*_78691 displayed a large reduction in editing, while expression of their editing factors OTP86 and CLB19 increased or remained stable, respectively. Finally, editing at *ndhD*_117166, which creates the AUG initiation codon of the *ndhD* transcript, is strongly inhibited under heat stress. Its editing requires the cooperative association between CRR4 and DYW1 (Boussardon *et al.* 2012; Kotera *et al.* 2005), and the latter shows a lower expression under heat stress. The NDHD protein is a subunit of the NADH dehydrogenase complex, which is postulated to have a role in plant stress acclimation (Rumeau *et al.* 2007). Increasing evidence now directly links RNA editing regulation to chloroplast development and stress responses, independently of editing factor regulation, and the wide range of behavior observed here under heat stress may be understood in this framework (Garcia-Andrade *et al.* 2013; Kakizaki *et al.* 2012; Zhang *et al.* 2014).

### Conclusions

We have presented ChloroSeq, a bioinformatics pipeline developed to investigate the chloroplast transcriptome systematically utilizing RNA-Seq data. Using ChloroSeq, information on chloroplast transcript accumulation, splicing, and editing can be determined simultaneously, and can be combined with nuclear transcript analysis. Although not shown here, ChloroSeq can also be used to study the mitochondrial transcriptome, which features some of same RNA-Seq analytical challenges as the chloroplast (Stone and Storchova 2015). Moreover, we see no reason why ChloroSeq could not be used outside of the plant kingdom. Most of the challenges posed by the derived organellar genomes of some unicellular eukaryotes, for example those of dinoflagellate chloroplasts (Howe *et al.* 2008), concern the alignment step, upstream of ChloroSeq. Once the alignment is performed, one can then use customized annotation files, making this pipeline easily adaptable to multiple research questions.

Here, we demonstrated the power of ChloroSeq through analysis of previously published datasets on abiotic stress, for which organellar data had not been studied. Among the stresses, heat stress was specifically associated with multifaceted effects on the chloroplast transcriptome. These included increased pncRNA accumulation, and decreases in splicing and editing efficiencies, all of which are phenomena that could potentially impact chloroplast protein synthesis. A global decrease in chloroplast protein synthesis following heat stress was earlier observed in *Phaseolus vulgaris* (Süss and Yordanov 1986). The main exception in that study was the PSII reaction center protein D1, which interestingly requires neither splicing nor editing for proper expression. A thorough review of the interaction of abiotic stresses with photosynthesis, and some of the response mechanisms, was recently published (Gururani *et al.* 2015), but the role of the chloroplast genome was not addressed. This highlights the tremendous opportunity ChloroSeq offers to gain knowledge of an essential participant in plant physiology, the chloroplast transcriptome.

## Supplementary Material

Supplemental Material
