# Supplementary material for: ChloroSeq, an Optimized Chloroplast RNA-Seq Bioinformatic Pipeline, Reveals Remodeling of the Organellar Transcriptome Under Heat Stress
Source: G3 (Bethesda). 2016 Jul 6;6(9):2817–27. doi: 10.1534/g3.116.030783 (PMC5015939; doi:10.1534/g3.116.030783)
Supplement: Supplemental Material [file supp_6_9_2817__index.html]

ChloroSeq, an Optimized Chloroplast RNA-Seq Bioinformatic Pipeline, Reveals Remodeling of the Organellar Transcriptome Under Heat Stress — Supplemental Material 

# ChloroSeq, an Optimized Chloroplast RNA-Seq Bioinformatic Pipeline, Reveals Remodeling of the Organellar Transcriptome Under Heat Stress

## Supplemental Material for Castandet *et al.*, 2016

**Files in this Data Supplement:**

- Figure S1 - Overview of chloroplast transcription under (a) heat, (b) cold, (c) salt and (d) drought stress. (.pptx, 1 MB)
- Figure S2 - Box plot representation of exon (a) and intron (b) RPKMs under different stress conditions. (.pptx, 85 KB)
- Figure S3 - Differences in gene expression for known splicing (a) and editing (b) factors after 3 and 12 h of heat stress. (.pptx, 165 KB)
- File S1 - Identification numbers for nuclear-localized genes encoding chloroplast-localized proteins involved in chloroplast RNA metabolism. (.txt, 4 KB)
