# Supplementary material for: ChloroSeq, an Optimized Chloroplast RNA-Seq Bioinformatic Pipeline, Reveals Remodeling of the Organellar Transcriptome Under Heat Stress
Source: G3 (Bethesda). 2016 Jul 6;6(9):2817–27. doi: 10.1534/g3.116.030783 (PMC5015939; doi:10.1534/g3.116.030783)
Supplement: Supplemental Material [file supp_g3.116.030783_FigureS1.pptx]

## Slide 1
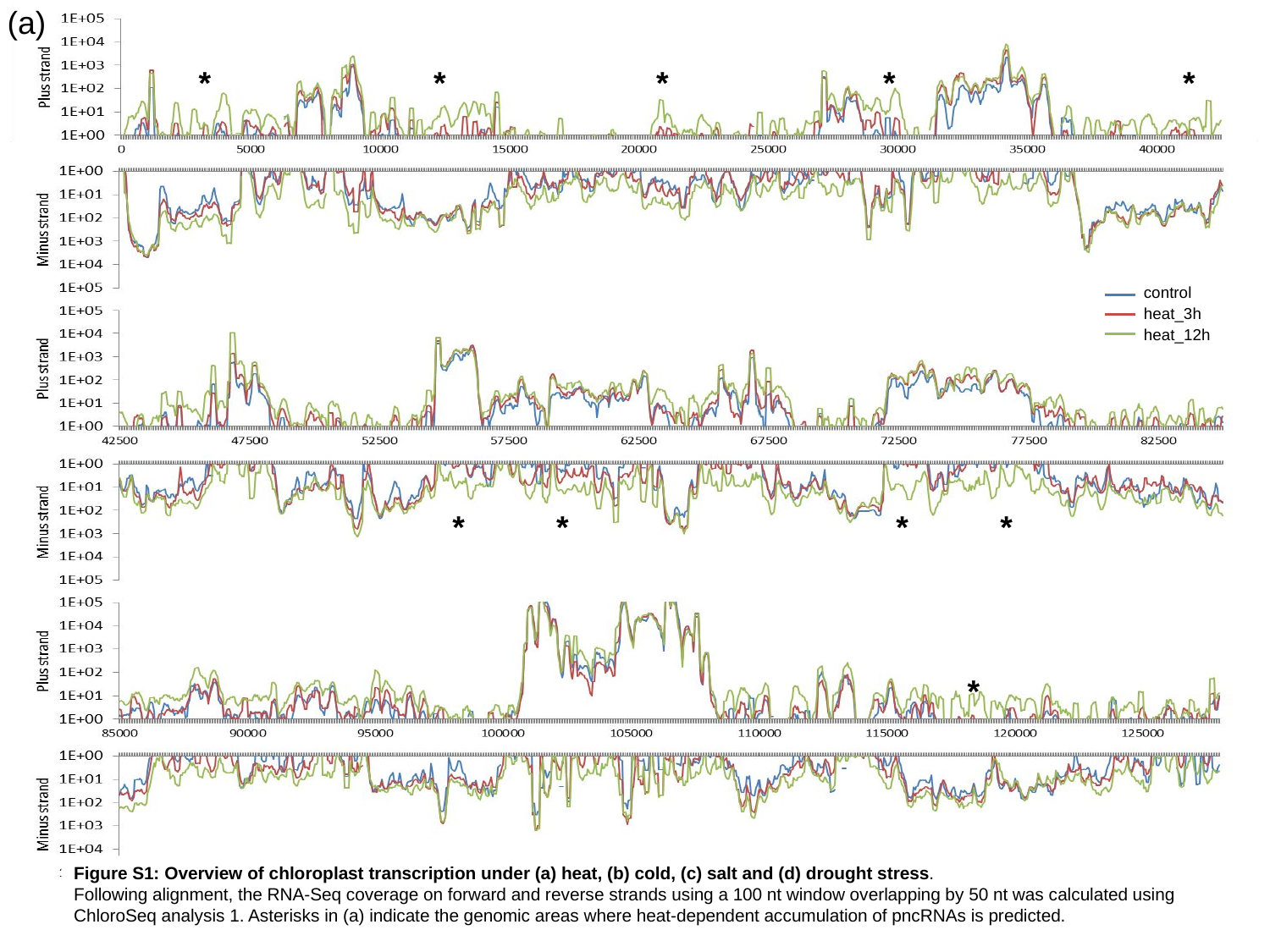

(a)
*
*
*
*
*
control
heat_3h
heat_12h
*
*
*
*
*
Supplementary figure 1A: Normalized sliding window of RNA-Seq coverage (reads) from plastid genome position 1 to 42500 for control and heat condition (37°C).
Following alignment, the coverage on both forward and reverse strand using a 100nt window overlapping by 50ntwas calculated using the get_coverage.sh
Figure S1: Overview of chloroplast transcription under (a) heat, (b) cold, (c) salt and (d) drought stress.
Following alignment, the RNA-Seq coverage on forward and reverse strands using a 100 nt window overlapping by 50 nt was calculated using ChloroSeq analysis 1. Asterisks in (a) indicate the genomic areas where heat-dependent accumulation of pncRNAs is predicted.

## Slide 2
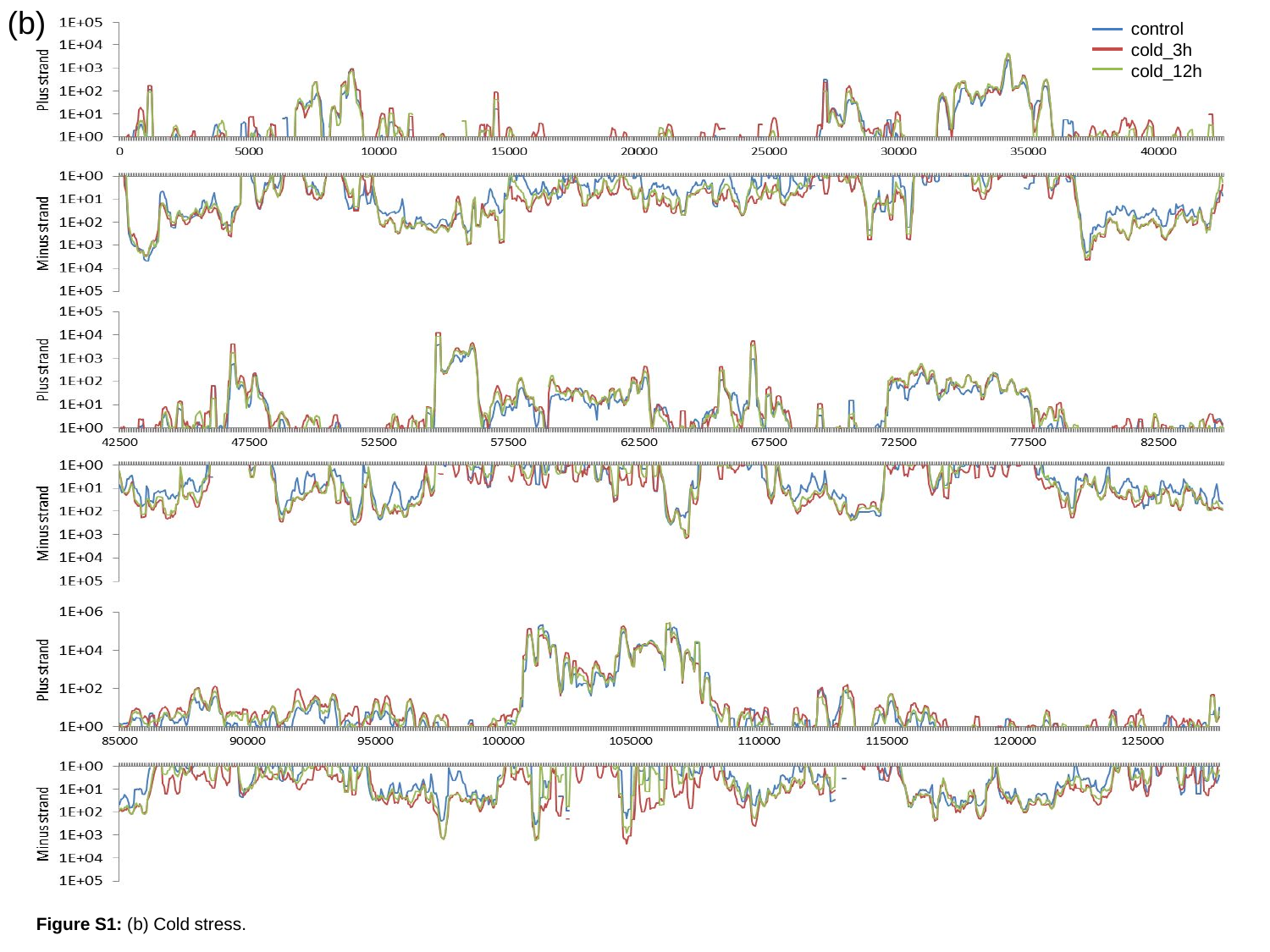

(b)
control
cold_3h
cold_12h
Figure S1: (b) Cold stress.

## Slide 3
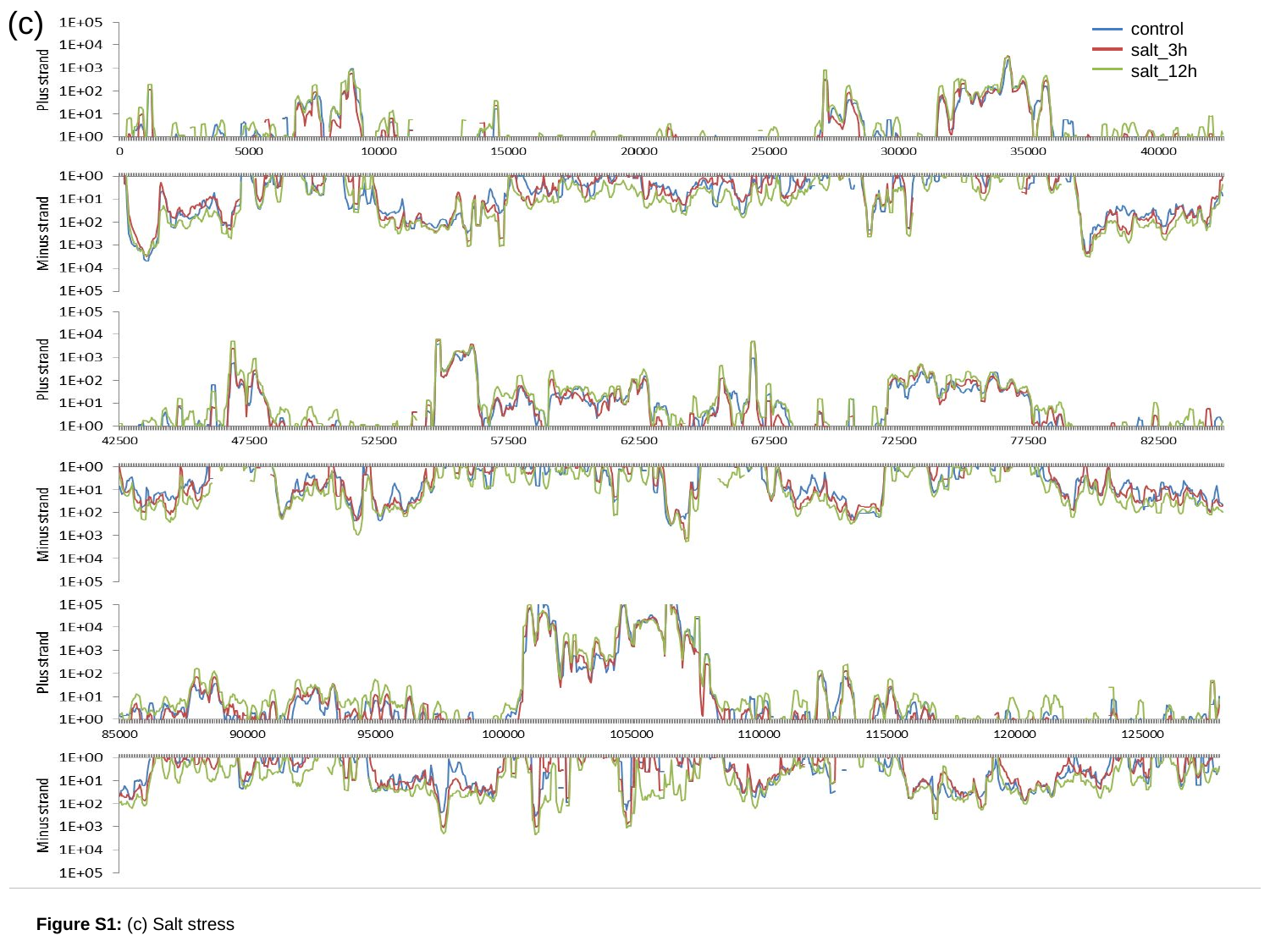

(c)
control
salt_3h
salt_12h
Figure S1: (c) Salt stress

## Slide 4
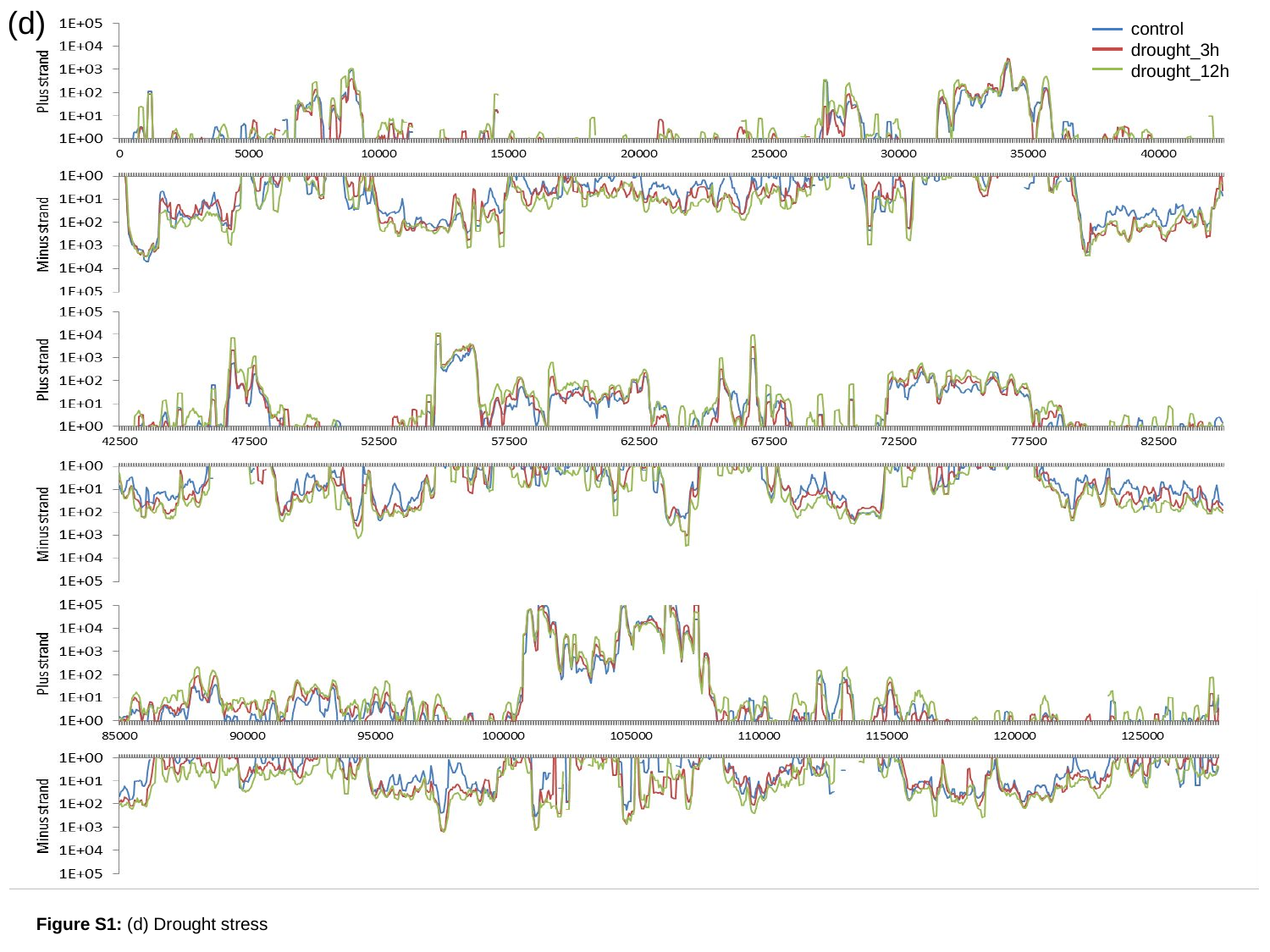

(d)
control
drought_3h
drought_12h
Figure S1: (d) Drought stress
