# Supplementary material for: ChloroSeq, an Optimized Chloroplast RNA-Seq Bioinformatic Pipeline, Reveals Remodeling of the Organellar Transcriptome Under Heat Stress
Source: G3 (Bethesda). 2016 Jul 6;6(9):2817–27. doi: 10.1534/g3.116.030783 (PMC5015939; doi:10.1534/g3.116.030783)
Supplement: Supplemental Material [file supp_g3.116.030783_FigureS2.pptx]

## Slide 1
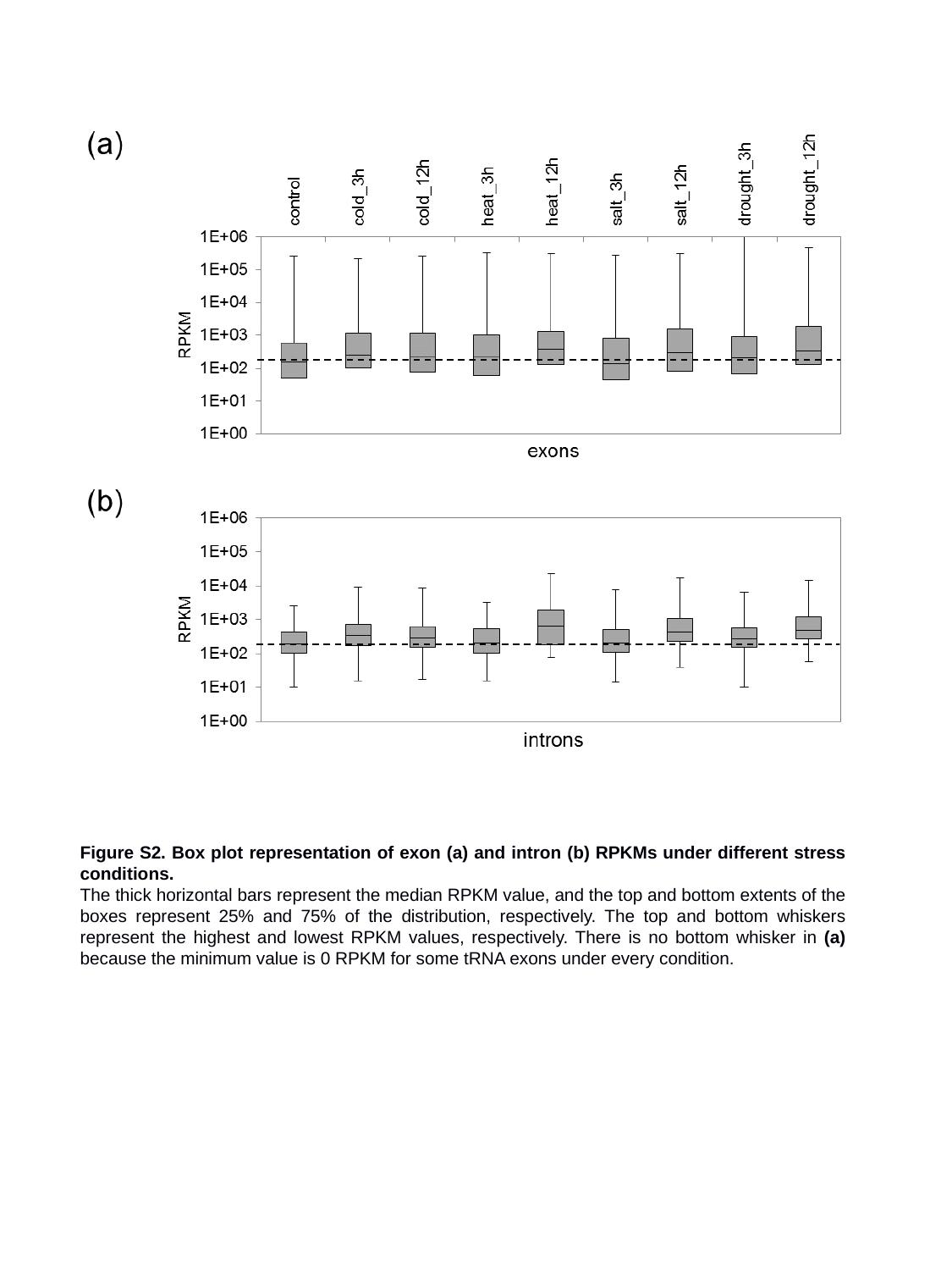

Figure S2. Box plot representation of exon (a) and intron (b) RPKMs under different stress conditions.
The thick horizontal bars represent the median RPKM value, and the top and bottom extents of the boxes represent 25% and 75% of the distribution, respectively. The top and bottom whiskers represent the highest and lowest RPKM values, respectively. There is no bottom whisker in (a) because the minimum value is 0 RPKM for some tRNA exons under every condition.
