# Supplementary material for: ChloroSeq, an Optimized Chloroplast RNA-Seq Bioinformatic Pipeline, Reveals Remodeling of the Organellar Transcriptome Under Heat Stress
Source: G3 (Bethesda). 2016 Jul 6;6(9):2817–27. doi: 10.1534/g3.116.030783 (PMC5015939; doi:10.1534/g3.116.030783)
Supplement: Supplemental Material [file supp_g3.116.030783_FigureS3.pptx]

## Slide 1
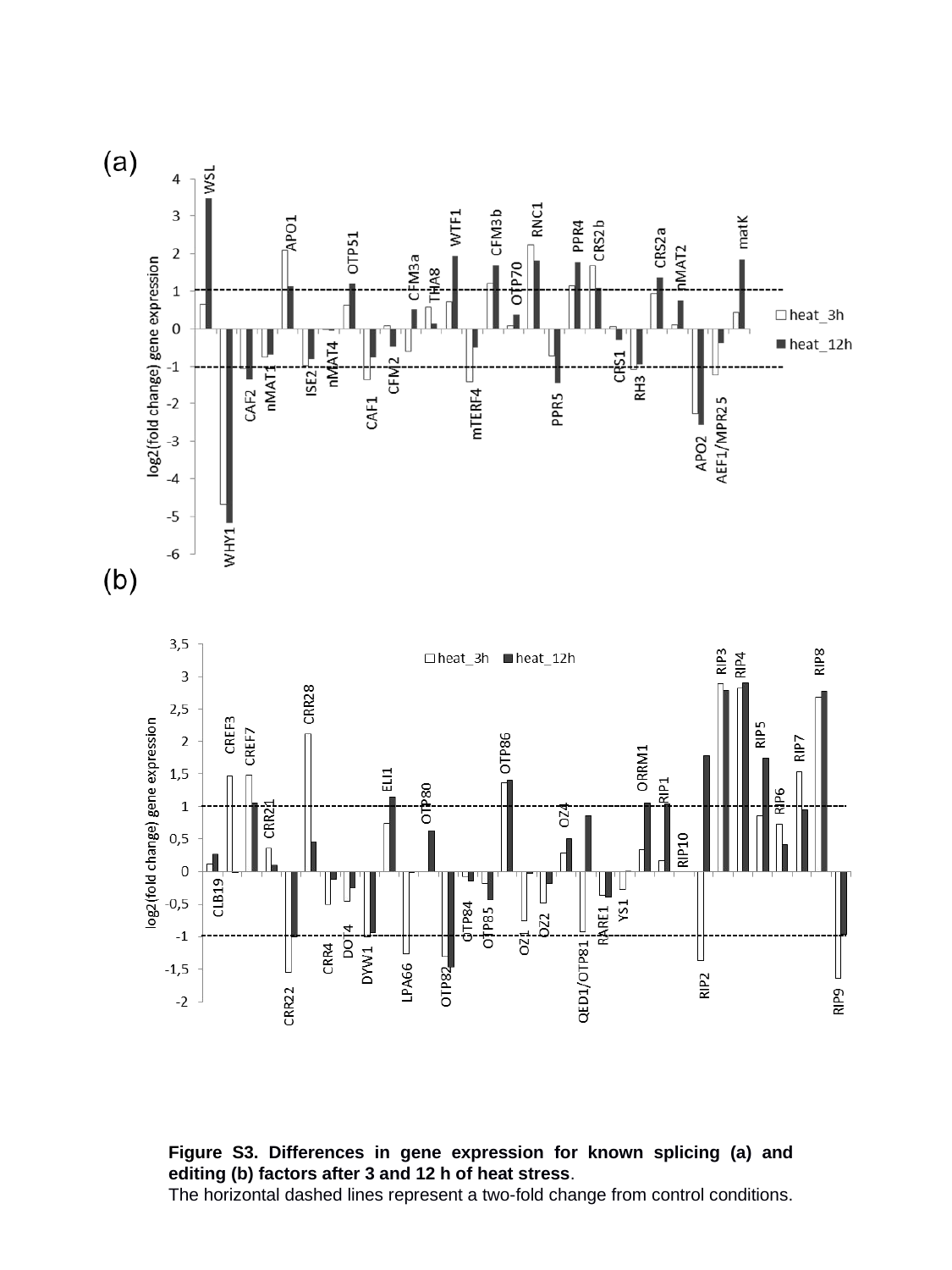

Figure S3. Differences in gene expression for known splicing (a) and editing (b) factors after 3 and 12 h of heat stress.
The horizontal dashed lines represent a two-fold change from control conditions.
